# Reducing Inequity in Urban Health: Have the Intra-urban Differentials in Reproductive Health Service Utilization and Child Nutritional Outcome Narrowed in Bangladesh?

**DOI:** 10.1007/s11524-018-0307-x

**Published:** 2018-08-29

**Authors:** Gustavo Angeles, Karar Zunaid Ahsan, Peter Kim Streatfield, Shams El Arifeen, Kanta Jamil

**Affiliations:** 1 Department of Maternal and Child Health, Gillings School of Global Public Health, University of North Carolina at Chapel Hill, Chapel Hill, NC USA; 2 Health System and Population Studies Division (HSPSD), International Centre for Diarrhoeal Disease Research, Bangladesh (icddr,b), Dhaka, Bangladesh; 3 Maternal and Child Health Division (MCHD), International Centre for Diarrhoeal Disease Research, Bangladesh (icddr,b), Dhaka, Bangladesh; 4 United States Agency for International Development (USAID)/Bangladesh, Dhaka, Bangladesh

**Keywords:** Bangladesh, Urban health, Slum, Health equity, Maternal health, Child nutrition, Family planning, Community health worker

## Abstract

Bangladesh is undergoing a rapid urbanization process. About one-third of the population of major cities in the country live in slums, which are areas that exhibit pronounced concentrations of factors that negatively affect health and nutrition. People living in slums face greater challenge to improve their health than other parts of the country, which fuels the growing intra-urban health inequities. Two rounds of the Bangladesh Urban Health Survey (UHS), conducted in 2013 and 2006, were designed to examine the reproductive health status and service utilization between slum and non-slum residents. We applied an adaptation of the difference-in-differences (DID) model to pooled data from the 2006 and 2013 UHS rounds to examine changes over time in intra-urban differences between slums and non-slums in key health outcomes and service utilization and to identify the factors associated with the reduction in intra-urban gaps. In terms of change in intra-urban differentials during 2006–2013, DID regression analysis estimated that the gap between slums and non-slums for skilled birth attendant (SBA) during delivery significantly decreased. DID regression analysis also estimated that the gap between slums and non-slums for use of modern contraceptives among currently married women also narrowed significantly, and the gap reversed in favor of slums. However, the DID estimates indicate a small but not statistically significant reduction in the gap between slums and non-slums for child nutritional status. Results from extended DID regression model indicate that availability of community health workers in urban areas appears to have played a significant role in reducing the gap in SBA. The urban population in Bangladesh is expected to grow rapidly in the coming decades. Wide disparities between urban slums and non-slums can potentially push country performance off track during the post-2015 era, unless the specific health needs of the expanding slum communities are addressed. To our knowledge, this is the first systematic explanation and quantification of the role of various factors for improving intra-urban health equity in Bangladesh using nationally representative data. The findings provide a strong rationale for continuing and expanding community-based reproductive health services in urban areas by the NGOs with a focus on slum populations.

## Introduction

Bangladesh is undergoing a rapid urbanization process. According to United Nations estimates [1], the urban population will grow from its 2015 level of 55 million people to 83 million in 2030, an increase of 51% in 15 years. Though it is largely a rural country (66% of the population lives in rural areas in 2015), all future population growth in the country is expected to be in urban areas, and Bangladesh will be an urban country by 2039 when the majority of people will live in urban areas. Dhaka is now the 11th largest mega city (defined as having more than 10 million inhabitants), with 17 million people, up from being 24th in the population ranking in 1990. Since Dhaka has one of the highest rates of population growth among mega cities (3.6% annually for 2010–2015), it is expected to become the world’s 6th largest city in 2030, with 27 million people [1].

Bangladesh has made impressive progress in reducing fertility and mortality and improving health and education conditions in recent decades [2], so rapid urbanization and urban health are now among the major population issues facing the country. It has been conjectured that urban poverty in Bangladesh is an area in urgent need of research and of new policy measures if the country is to meet the national goals of poverty reduction [3]. Urban populations are characterized by large inequalities in economic, living, and health-related conditions. The heterogeneity of urban settings is primarily fueled by the migration process, where most rural migrants have few resources (financial and otherwise) when they arrive in cities. There they are faced with inadequate infrastructure and public services, as well as tight formal labor markets, which results in growing intra-city inequality and urban poverty [4–6]. An expression of this inequality is the coexistence in cities of well-developed areas alongside slums, which are areas of dense concentrations of people and of conditions that affect health negatively. At present, it is estimated that one-third of Bangladesh’s City Corporations’ population lives in slums [7]. Earlier studies have established that people living in the slum settlements experience social, economic, and political exclusion [8] and have weak social network [9, 10] which bars them from society’s basic resources. People living in urban slums face much greater challenge to improve their health than other parts of the country, which fuels the growing intra-urban health inequities despite living in relatively close proximity to health service [11].

The National Sustainable Development Strategy 2010–2021 of the Government of Bangladesh identifies urban development as one of the five strategic priority areas for sustainable development [12]. The government is cognizant of the fact that urban areas are now afflicted with innumerable problems ranging from lack of provision of services to deteriorating environmental conditions [13]. The recently approved 7th Five Year Plan for 2016–2020 also places focus on improving the conditions of urban populations and set a number of specific actions and coordination mechanism to reduce urban inequity in the coming years [14].

As regards health service delivery in the urban area—there are gaps in service coverage from the public sector—urban poor are particularly vulnerable due to limited government healthcare infrastructure and provisions within their reach [13, 14] and a host of other actors like community-based organization, non-government organizations (NGOs), and private sector dominates health services provision in urban Bangladesh [14]. Over the past two decades, provision of primary health care in urban areas has been built through a partnership between the government, development partners, and NGOs—with support from development partners like Asian Development Bank, government contracted NGOs for the delivery of basic health services [15, 16]. However, the share of private sector for health services in the urban areas is 80%, which overwhelmingly dominated by pharmacies and non-formal or traditional doctors [17].

In this context, understanding performance of major urban domains in terms of health outcomes and healthcare utilization is crucial for planning and policy making. The purpose of this paper is to assess whether intra-urban differentials in health and care use between slums and non-slums have narrowed between 2006 and 2013 in the main cities of Bangladesh and to identify the factors associated with the change in intra-urban gaps.

## Materials and Methods

Despite the recognized importance of examining intra-urban inequalities, there have been few datasets based on representative samples to support that analysis. For this reason, in 2006, the first Urban Health Survey (UHS) was conducted with separate sampling domains for slums and non-slum areas within Dhaka and five city corporations (Chittagong, Khulna, Rajshahi, Sylhet, and Barisal) and in district municipalities to identify the health challenges and use of services of key subpopulations of the cities. The UHS 2013 is a follow-up survey conducted after 7 years to examine the changes in the health and service utilization profile of the urban population in Dhaka and eight city corporations (Chittagong, Khulna, Rajshahi, Sylhet, Barisal, Rangpur, Narayanganj, and Comilla) and in district municipalities and large towns with populations over 45,000 habitants as listed in the most recent National Population Census (this domain was referred as “other urban areas” in the UHS 2013). We used data from these two high-quality and highly comparable household surveys for the primary analyses in this study. Both surveys were large, covering about 12,069 households in 2006 and 53,790 households in 2013, and they are representative of the urban slums and non-slums domains as the sampling design was based on a sampling frame prepared from a special Census and Mapping of Slums conducted in 2005 [18, 19]. Both surveys obtained information about selected background characteristics, a full birth history from ever-married women aged 13–49 years to provide indicators on maternal and child health, health-seeking behaviors, and intervention coverage.

Using pooled data from 2006 and 2013 rounds of UHS for Dhaka and five city corporations, we carried out bivariate and multivariate analyses of selected health outcomes and healthcare utilization indicators in urban slums and non-slums to examine how the intra-urban inequity has changed over this period. The outcomes of interest and variables selected for the analyses are presented in Table 1. The dependent variables considered in this analysis are facility delivery (whether the last birth within 3 years preceding the survey was delivered in a health facility), use of modern family method (by currently married women), and stunting among under-five children (inadequate length/height for age according to the World Health Organization’s standard growth, which is reflects long-term effects of undernutrition). The independent variables/covariates used in the analysis are as follows: mother’s educational attainment (categories based on years of completed schooling); length of residence (categories based on duration in years in city of current residence); region (whether the respondent lives in the country’s capital, Dhaka); socioeconomic status (proxy of socioeconomic status based on possession of household assets); distance to the nearest health facility (categories based on distance in kilometer); availability of community health worker; birth order (categories based on parity); and mother’s age at birth (categories based on age in years).

**Table 1:** Dependent variables and selected determinants for analysis.

Dependent variables	Independent variables		
	Structural	Programmatic	Demographic
Facility delivery	Mother’s educational attainment	Distance to the nearest health facility	Birth order
Use of modern family planning methods	Length of stay in city of current residence	Community health worker availability	Mother’s age at birth
Stunting among under-5 children	Region		
	Socioeconomic status		

To examine changes over time in the gaps between slums and non-slums, we need a model that can present, in a unified framework, the difference between slum and non-slum in 2006 (the 2006 gap), the difference in 2013 (the 2013 gap), and that it can compare the differences to test if the gaps are reducing over time. We used an adaptation of the difference-in-differences (DID) model, applied to pooled data from the 2006 and 2013 UHS, to do precisely that. The basic DID model is:

1 $$ {Y}_{ijt}={\alpha}_0+{\alpha}_1{S}_{jt}+{\alpha}_2{T}_t+{\alpha}_3{S}_{jt}\cdot {T}_t+{\alpha}_4{X}_{ijt}+{\varepsilon}_{ijt} $$

where *Y*_*ijt*_ is the outcome of interest for individual *i* who lives in community *j* at time *t*. *S*_*jt*_ takes the value of 1 if community *j* is a slum and 0 if it is a non-slum. *T*_*t*_ is an indicator variable that takes the value of 1 if the observation corresponds to year 2013 UHS and 0 if the observation was taken in the 2006 UHS. *S*_*jt*_ ∙ *T*_*t*_ is the interaction term of the previous two indicator variables, and *ε*_*ijt*_ is the error term that represents unobserved sources of variation in the outcome. The parameter of interest is *α*_3_, the coefficient of the interaction term, which measures the change in the gap between slums and non-slums in the outcome that occurred between 2006 and 2013. The model can easily accommodate covariates (*X*_*ijt*_) with factors that influence the outcomes.

After examining the changes over time in the gaps between slums and non-slums in selected outcomes, we extended the basic DID model to identify the factors associated with the reduction in intra-urban inequities, as observed from (1). Out of the determinants considered in the basic DID model (outlined in Table 1), the extended model aims to explore (a) if the intra-urban gap narrowed more in Dhaka than in the other city corporations and (b) if comprehensive CHW coverage was associated with lowering the intra-urban gap. The extended DID model looks like:

2 $$ {\displaystyle \begin{array}{l}{Y}_{ijt}={\beta}_0+{\beta}_1{S}_{jt}+{\beta}_2{T}_t+{\beta}_3{S}_{jt}\cdot {T}_t+{\beta}_4{Z}_{ijt}\\ {}\kern2em +{\beta}_5{Z}_{ijt}\cdot {S}_{jt}+{\beta}_6{Z}_{ijt}\cdot {T}_t+{\beta}_7{Z}_{ijt}\cdot {S}_{jt}\cdot {T}_t+{\beta}_8{X}_{ijt}+{\varepsilon}_{ijt}\end{array}} $$

where *Z*_*ijt*_ is an indicator variable that takes the value of 1 if individual *i* lives in Dhaka (or lives in a community where services from CHW are available) and 0 if the individual lives in other city corporation (or lives in a community where services from CHW are not available). The other variables have the same definition as before. In this model, *β*_3_ measures the change in the slums to non-slums gap that occurred between 2006 and 2013 in other city corporations (or in communities without CHW), and (*β*_3_ + *β*_7_) measures the equivalent change that occurred in Dhaka (or in communities with CHW). By examining these two measures, we can determine if the gap narrowed more in Dhaka or in the other city corporations (or if the gap narrowed more in communities with or without CHWs).

We estimate (1) and (2) using a linear probability model (LPM) [20]. The advantage of LPM is the ease of interpreting the estimated parameters [21]. We used sampling probability weights and control for clustering and heteroscedasticity to obtain robust standard errors.

## Results

### Changes in Background Characteristics of Urban Bangladesh During 2006–2013

In 2013, currently married women living both in slums and non-slums have become slightly older, wealthier, and more educated and have borne fewer children than in 2006 (see Table 2). However, currently married women living in slums are markedly younger, poorer, and less educated and have more children than their non-slum counterparts.

**Table 2:** Distribution (%) of structural, programmatic, and demographic characteristics among currently married women by slum and non-slum areas in urban city corporations of Bangladesh, 2006 UHS and 2013 UHS

Background characteristics	Non-slums		Slums	
	2006	2013	2006	2013
Parity				
0	12.8	11.1	11.7	11.2
1	20.8	26.1	20.4	25.0
2	30.0	34.0	21.6	26.4
3+	36.5	28.8	46.3	37.4
Woman’s age				
< 20 years	6.8	5.4	13.0	9.1
20–34 years	60.5	57.5	59.7	62.7
35+ years	32.6	37.1	27.3	28.2
Region				
Dhaka	53.5	66.4	64.0	64.4
Other division	46.5	33.6	35.9	35.6
Mother’s education				
No education	20.2	9.8	46.8	29.7
Primary incomplete	10.5	10.2	16.6	24.1
Primary complete	12.3	9.0	13.4	15.2
Secondary incomp.	27.1	30.7	18.4	24.9
Secondary +	29.9	40.3	4.8	6.1
Length of stay in the current city of residence				
< 2 years	6.4	6.5	10.8	8.8
2–4 years	11.1	8.9	12.8	11.9
5+ years	49.2	44.7	49.2	48.7
Lived always	33.2	39.9	27.2	30.7
Household’s asset ownership				
< 2 items	12.6	6.0	55.1	41.0
2 items	22.7	14.1	27.6	32.5
3–4 items	64.5	79.9	17.3	26.5
Distance from health facility				
< 1 km	74.3	74.1	65.4	70.7
1–2 km	18.0	21.7	22.6	24.4
> 2 km	7.7	4.2	12.0	4.9
Number of available community health worker				
None	87.3	49.2	78.2	35.2
One	6.1	21.0	15.0	23.8
Two or more	6.6	29.8	6.8	40.9
Observations	3993	6790	5128	11,974

To ensure comparability, same city corporations were considered from both the survey rounds

### Changes in Selected Health Outcomes in Urban Bangladesh During 2006–2013

#### Family Planning (FP)

**Fig. 1:**
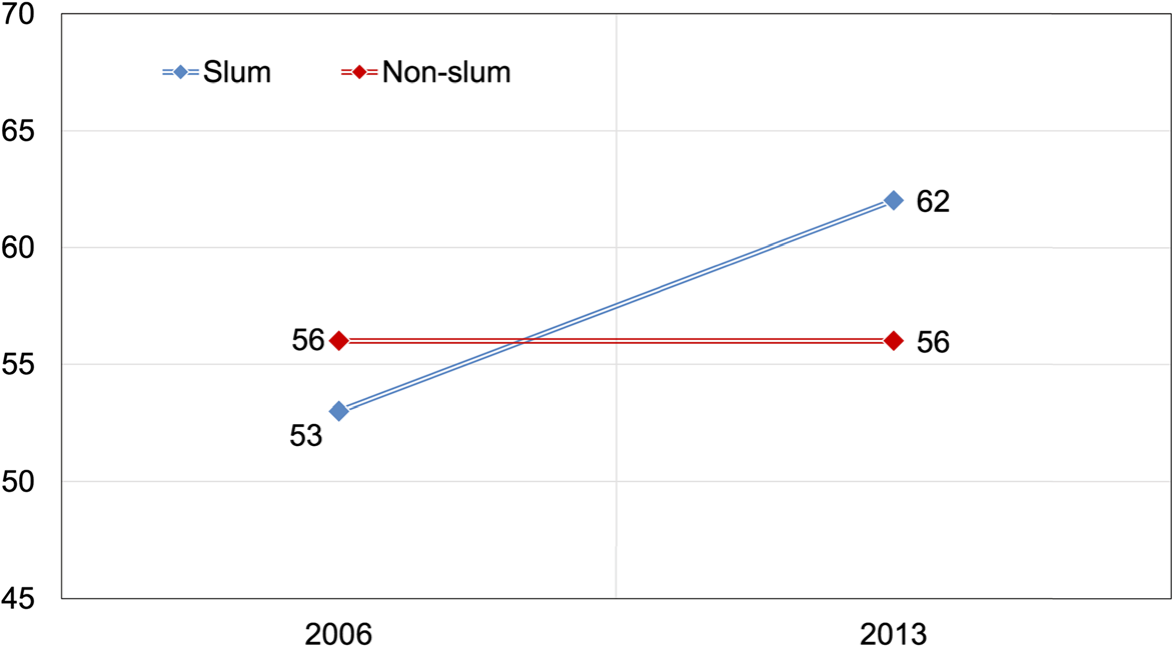
Trends in modern contraceptive use in slums and non-slums, 2006 and 2013. Percent of currently married women of age 15–49 years using a modern contraceptive method during the time of the survey

Family planning performance in slums surpassed that in non-slums between 2006 and 2013. As seen in Fig. 1, contraceptive prevalence rate (CPR) for modern methods remained unchanged at 56% in non-slum areas over the 7 years between surveys, whereas it increased by 9 percentage points in slums between 2006 and 2013, to reach 62%, an impressive increase that reversed the intra-urban differential.

#### Skilled Birth Attendant (SBA) During Delivery

**Fig. 2:**
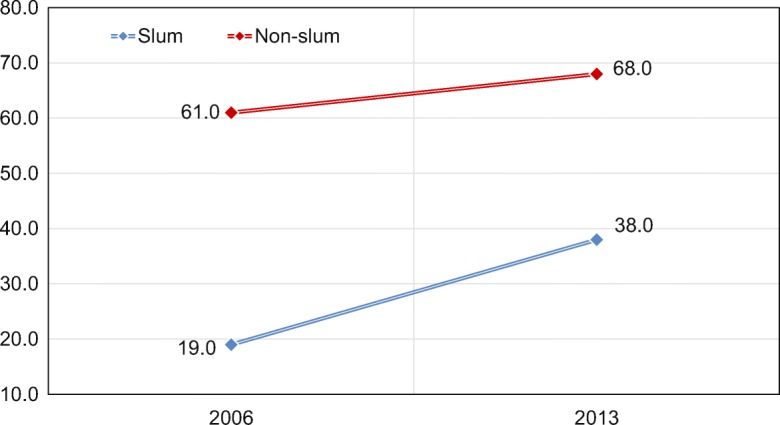
Trends in deliveries by skilled birth attendant in slums and non-slums, 2006 and 2013. Percent of live births in the 3 years preceding the survey assisted by a medically trained provider, which includes qualified doctor, nurse/midwife, paramedics, Family Welfare Visitor (FWV), community-based skilled birth attendant (CSBA), and Medical Assistant/Sub-assistant Community Medical Officer (MA/SACMO)

Between 2006 and 2013 the intra-urban differential in use of SBA declined (Fig. 2). In 2006, births among women living in non-slums were 3.2 times more likely to be assisted by a medically trained provider compared to births among women living in slums. This ratio declined to 1.8 in 2013. Also, the absolute difference in SBA between slums and non-slums declined from 42 percentage points to 30 percentage points in the last 7 years.

#### Child Nutritional Status

**Fig. 3:**
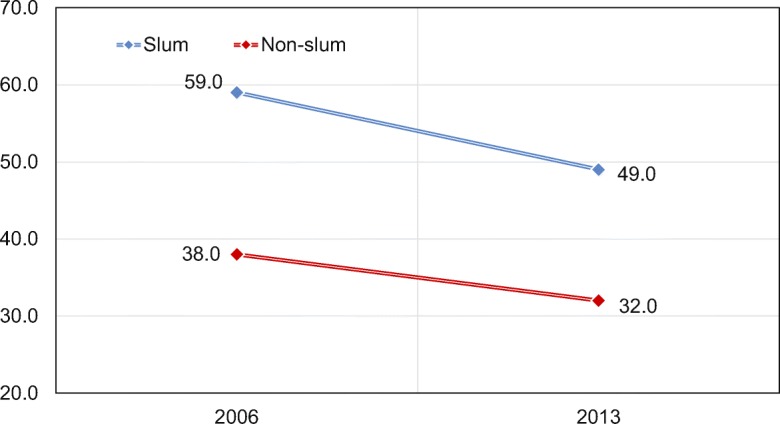
Trends in stunting among children in slums and non-slums, 2006 and 2013. Percent of children under 5 years classified as stunted, i.e., having height for age below − 2 SD

The intra-urban difference in nutritional status (defined as moderate or severe stunting among under-five children) among children improved only marginally, by 3 percentage points, during 2006–2013 (Fig. 3). One in two children living in slums and one in three children living in non-slums were stunted in 2013, indicating that the prevalence of stunting was 1.53 times higher in slum than in non-slum populations. That ratio was almost unchanged from 2006 (1.55). It is worth mentioning that the level of stunting registered in rural areas in 2014 was 37.9%, a much lower level than registered in slums [22, 23].

### Did the Intra-urban Gap in Key Outcomes Narrow Between 2006 and 2013?

Results from the DID regression analysis indicate that for SBA, the gap between slums and non-slums narrowed significantly (by 11.5 percentage points, at *p* < 0.05) during 2006–2013 (see the coefficient of the interaction “slum × 2013” in model 1 simple DID under SBA in Table 3). Even after controlling for structural, programmatic, and demographic characteristics, separately and in combination, the reduction in the gap between slums and non-slums remains statistically significant and about similar magnitude, in the range of 10.6 to 11.5 percentage points (see models 2–5 under SBA in Table 3).

**Table 3:** Regression estimates of structural, programmatic and demographic factors on selected outcomes in urban city corporations of Bangladesh between 2006 and 2013

Variables	SBA during delivery					Modern FP use					Under-5 stunting				
	Model 1 simple DID	Model 2	Model 3	Model 4	Model 5	Model 1 simple DID	Model 2	Model 3	Model 4	Model 5	Model 1 simple DID	Model 2	Model 3	Model 4	Model 5
Parity (ref: parity 0)															
1				ref	ref				0.357***	0.357***				ref	ref
2				− 0.111***	− 0.064***				0.507***	0.511***				0.038**	0.018
3+				− 0.208***	− 0.093***				0.522***	0.538***				0.081***	0.031
Mother’s age (ref: < 20 years)															
20–34 years				0.134***	0.059**				− 0.104***	− 0.010***				− 0.075***	− 0.046**
35+ years				0.189***	0.126**				− 0.342***	− 0.330***				− 0.086**	− 0.063
Mother’s educational attainment (ref: no education)															
Primary inco.		0.021			0.020		0.092***			0.080***		− 0.010			− 0.012
Prim. compl.		0.021			0.015		0.081***			0.078***		− 0.042*			− 0.044*
Secondary		0.143***			0.132***		0.050***			0.066***		− 0.094***			− 0.095***
Secondary+		0.386***			0.363***		0.053***			0.097***		− 0.160***			− 0.151***
Length of stay in the current city of residence (ref: < 2 years)															
2–4 years		0.034			0.031		0.062***			0.019		0.003			0.002
5+ years		0.049**			0.057**		0.081***			0.005		− 0.006			− 0.005
Lived always		0.148***			0.150***		0.052***			− 0.004		0.015			0.014
Region (ref: outside Dhaka)															
Dhaka		0.035**			0.027*		− 0.010			− 0.010		0.030**			0.029**
Socioeconomic status (ref: household owns < 2 items)															
2 items		0.078***			0.075***		− 0.009			0.011		− 0.075***			− 0.073***
3–4 items		0.224***			0.220***		− 0.060***			− 0.029**		− 0.099***			− 0.098***
Number of available community health worker (ref: none)															
One			− 0.002		0.016			− 0.002		− 0.005			− 0.004		− 0.016
Two or more			0.050**		0.045**			0.005		0.006			0.023		0.016
Distance from health facility (ref: < 1 km)															
1–2 km			− 0.028*		− 0.016			0.005		− 0.0004			− 0.010		0.015
> 2 km			− 0.071**		− 0.057**			− 0.011		− 0.014			0.045		0.043*
Urban domain (ref: city corporation non-slums)															
CC slum	− 0.414***	− 0.161***	− 0.410***	− 0.388***	− 0.160***	− 0.022	− 0.035*	− 0.021	− 0.048**	− 0.037*	0.194***	0.098***	0.194***	0.183***	0.098***
Survey round (ref: 2006)															
2013	0.072**	.021	0.059*	0.055*	0.001	0.007	0.014	0.006	0.020	0.017	− 0.060**	− 0.037	− 0.062**	− 0.049*	− 0.032
Interaction between urban domain and survey round															
Slum × 2013	0.115**	0.115***	0.106**	0.112**	0.108**	0.082***	0.068***	0.081***	0.081***	0.071***	− 0.030	− 0.031	− 0.032	− 0.033	− 0.034
Constant	0.608***	0.162***	0.616***	0.596***	0.182***	0.556***	0.489***	0.556***	0.314***	0.256***	0.385***	0.529***	0.382***	0.406***	0.546***
Observations	7536	7536	7536	7536	7536	27,885	27,885	27,885	27,885	27,885	9353	9353	9353	9353	9353

*p < 0.10, **p < 0.05, ***p < 0.001

For use of modern FP methods by currently married women of age 15–49 years, DID regression analysis estimated that the gap between slums and non-slums narrowed significantly (by 8.2 percentage points, at *p* < 0.001) during 2006–2013 (see the coefficient of the interaction “slum × 2013” in model 1 simple DID under modern FP use in Table 3). Indeed, the gap reversed in favor of slums. Even after controlling for structural, programmatic, and demographic characteristics, separately and in combination, the reduction in the gap between slums and non-slums remains statistically significant and in the range of 6.8 to 8.1 percentage points (see models 2–5 under modern FP use in Table 3).

However, in terms of moderate or severe stunting level among children under age 5 years, the DID estimates indicate a small but not statistically significant reduction in the gap between slums and non-slums during 2006–2013 (see models 1–5 under under-5 stunting in Table 3).

### Was the Reduction of the Intra-urban Gap Greater in Dhaka or in the Other City Corporations?

Dhaka City Corporation dominates the total urban population in Bangladesh and continues to increase at a high rate of population growth to become the world’s 6th largest city in 2030 [1]. This warrants special emphasis on Dhaka to review whether healthcare utilization in this city corporation evolving differently than the rest of the city corporations. Results from the extended DID model indicate that for SBA, the reduction of the intra-urban gap in Dhaka was of 12.2 percentage points and in the other city corporations was of 11.4 percentage points. While both reductions were statistically significant at the 5% level, the difference between them was not, indicating that similar declines in the gap occurred and Dhaka and the other city corporations (see model 1 under SBA in Table 4). Similar results were obtained for modern contraceptive use: the decline of the intra-urban gap was statistically significant at 7.2 percentage points in Dhaka, while in other city corporations was of 9.2 percentage points and significant. The difference of the reductions was not statistically significant (see model 1 under modern FP use in Table 4).

**Table 4:** Regression estimates of the effect of selected factors in reducing intra-urban difference in Bangladesh between 2006 and 2013

Variables and interactions	SBA during delivery		Modern FP use	
	Model 1	Model 2	Model 1	Model 2
Slum domain (ref: non-slums)				
Slum	− 0.399*** (− 0.478, − 0.320)	− 0.407*** (− 0.478, − 0.335)	− 0.014 (− 0.075, 0.047)	− 0.028 (− 0.071, 0.015)
Survey round (ref: 2006)				
2013	0.075* (− 0.009, 0.160)	0.075** (0.002, 0.148)	− 0.018 (− 0.078, 0.042)	− 0.004 (− 0.045, 0.036)
Interaction between slum and survey round				
Slum × 2013	0.114** (0.011, 0.218)	0.066 (− 0.027, 0.159)	0.092** (0.024, 0.161)	0.103*** (0.052, 0.154)
Region (ref: other city corporations)				
Dhaka	0.041 (− 0.068, 0.150)		− 0.025 (− 0.090, 0.040)	
Interaction between Dhaka and slum				
Dhaka × slum	− 0.032 (− 0.154, 0.090)		− 0.008 (− 0.082, 0.066)	
Interaction between Dhaka and survey round				
Dhaka × 2013	− 0.011 (− 0.134, 0.112)		0.042 (− 0.028, 0.113)	
Interaction among Dhaka, slum, and survey round				
Dhaka × slum × 2013	0.008 (− 0.139, 0.156)		− 0.020 (− 0.103, 0.063)	
Availability of community health worker (ref: CHW not available)				
CHW available		0.036 (− 0.083, 0.155)		− 0.018 (− 0.082, 0.046)
Interaction between CHW availability and slum				
CHW × slum		− 0.051 (− 0.186, 0.084)		0.037 (− 0.036, 0.110)
Interaction between CHW availability and survey round				
CHW × 2013		− 0.032 (− 0.163, 0.099)		0.036 (− 0.032, 0.105)
Interaction among CHW availability, slum, and survey round				
CHW × slum × 2013		0.115 (− 0.042, 0.272)		− 0.064 (− 0.146, 0.018)
Constant	0.586*** (0.514, 0.658)	0.603*** (0.540, 0.666)	0.570*** (0.513, 0.626)	0.558*** (0.522, 0.595)
Addition of main interaction terms	0.122** (0.017, 0.228)	0.181** (0.054, 0.308)	0.072** (0.025, 0.119)	0.039 (− 0.025, 0.104)
Observations	7536	7536	27,885	27,885

*p < 0.10, **p < 0.05, ***p < 0.001

### Was the Reduction of the Intra-urban Gap Greater in Areas with CHWs?

Given the gaps in the provision of health services in the urban areas [13, 14], review of the roles of community-based organizations and CHWs in increasing health services provision is crucial. Results from the extended DID model indicate that the expansion of CHWs in urban areas during 2006–2013 appears to have played a significant role reducing the gap in SBA. Areas with CHWs had a significant reduction of 18.1 percentage points in the intra-urban gap, while areas with no CHW show a smaller decline of the gap, in 6.6 percentage points, but it is not statistically significant (see model 2 under SBA in Table 4). However, for modern family planning use, the results are reversed: a large significant decline of 10.3 percentage points in the intra-urban gap in modern FP use is estimated in areas with no CHW, while in areas with CHW, the reduction is small and not statistically significant (see model 2 under modern FP in Table 4).

## Discussions

The findings from the DID regression analyses demonstrated that the overall intra-urban difference in use of modern FP methods and SBA significantly narrowed during 2006–2013, meaning the healthcare utilization of the people living in the city corporation slums are improving at a faster rate than their non-slum counterparts. The key question is what has been happening differently in slums that could explain this remarkable change in family planning and maternal health services in urban Bangladesh?

Unlike the rural areas of Bangladesh, where the public sector has a comprehensive infrastructure to deliver FP services up to the doorstep through the agencies under the Ministry of Health and Family Welfare (MOHFW) [22], FP service delivery channels are different in urban areas—there is no particular public sector infrastructure for FP service delivery in the city corporations [14]. Selected NGO and private clinics provide intrauterine device (IUD), implants, and female and male sterilizations along with public sector medical college hospitals and general/district hospitals that were located in the city corporations. Delivery of primary health care (PHC) services, primarily comprising reproductive and child health and family planning services, in urban areas is the responsibility of the Ministry of Local Government, Rural Development and Cooperatives (MOLGRDC), which have been implementing PHC projects through NGOs [24]. In addition, a number of major development partners support reproductive and child health services in urban areas that are also implemented by NGOs.

**Fig. 4:**
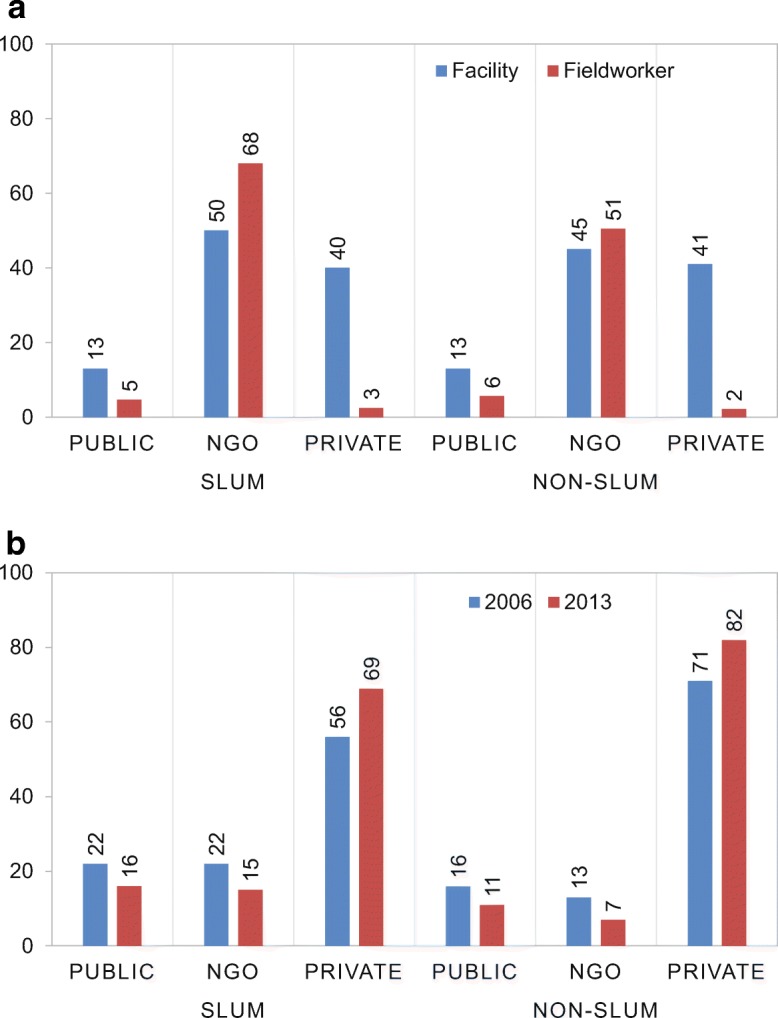
**a** Availability of facilities and fieldworkers in slums and non-slums, 2013. Percent of surveyed clusters by availability of health facility within specified distance or a community health worker. **b** Trends in sources for contraceptive methods in slums and non-slums, 2006 and 2013. Percent of users of modern contraceptive methods ages 15–49 by the most recent source of supply

Findings from 2006 and 2013 UHS indicated an appreciable improvement in the living conditions of slum dwellers in terms of housing structures and household possessions [18, 19]. As economic conditions are improving, it appears that people are willing to pay for reproductive health services, in particular family planning services—despite the greater coverage of NGO sector in terms of health facilities and health/FP fieldworkers both in slums and non-slums (see Fig. 4a), only the private sector’s share increased between 2006 and 2013 as the source for contraceptives—the rate of increase is more pronounced among contraceptive users living in slums (see Fig. 4b). Earlier studies also found that urban people in Bangladesh are generally willing to pay for family planning services from NGO or private providers where private clinics compete against the government facilities providing free services [25]. More recent cross-sectional surveys in large cities of Bangladesh found that even the majority of socioeconomically vulnerable population groups like slum residents and street-dwellers were willing to pay for reproductive health services [26, 27].

In terms of methods mix, CPR for pills, condom, and injectables (comprising nearly 90% of CPR for modern methods both in slums and non-slums) increased during 2006–2013, whereas in the city corporation non-slums, CPR for only condoms increased [18, 19]. One reason for over-reliance on short-acting contraceptive methods could be the frequent use of the private sector, overwhelmingly comprising the drug stores or pharmacies, where pills and condoms are available at affordable prices. This (over-reliance on short-acting methods purchased from drug stores/pharmacies) may partly explain the significant, faster decline in intra-urban gap in modern FP use in areas with no CHWs as indicated by extended DID analysis. Further study is required to look into the underlying population characteristics of city corporation slums with no CHW coverage, along with thorough assessment of CHW capacity and quality of FP services in areas with CHWs to identify the reasons for faster decline in intra-urban gap in modern FP use in areas with no CHWs. Further studies are also required to look into the stalling of contraceptive use in non-slums areas to better understand their fertility preferences and need to fertility regulation.

Maternal healthcare utilization (e.g., SBA) has also markedly increased in slums, significantly faster than in non-slums, during 2006–2013. However, maternal healthcare indicators still remain poorer in the slums than in non-slums, and antenatal care (ANC) and post-natal care (PNC) rates are about half in the slums compared to non-slums [18]. Outside secondary- and tertiary-level facilities like general hospitals/medical college hospitals, urban areas lack the primary-level public facilities under the MOHFW to provide maternity care. This gap is filled by private clinics and hospitals and NGO health facilities.

The MOLGRD&C runs 138 clinics through NGOs known as Comprehensive Reproductive Healthcare Centre (CRHCC) and Primary Healthcare Centre (PHCC) to provide maternal health services in urban areas. The USAID- and DFID-funded NGO Health Service Delivery Project (NHSDP) operates 207 static clinics in the urban areas that provide an essential package of health services that includes family planning, maternal, newborn, and child health services through a network of local NGOs. Under NHSDP, free and subsidized services are also provided through “Smiling Sun” static clinics, satellite clinics, and CHWs. Marie Stopes Bangladesh provides a range of maternal health and family planning services through 141 clinics in urban and peri-urban areas, out of which 15 are maternities that conduct deliveries, including C-section. Manoshi is a community-based health program implemented by BRAC to provide maternal, neonatal, and child health services to urban slum dwellers in 10 cities in Bangladesh. Using two levels of CHWs, Manoshi provides ANC and PNC through home visits and delivery care through 29 clinics/maternity centers in urban areas [28]. As CHWs play a crucial role in delivering reproductive health services in urban areas [29, 30], the role of CHWs appears to have a positive impact in reducing intra-urban inequity in SBA utilization, as the results from extended DID analysis indicated.

Unlike contraceptive prevalence or skilled assistance during delivery, the intra-urban difference in nutritional status among under-five children did not reduce significantly—slum dwellers still had over 50% higher levels of child malnutrition than non-slum dwellers, and the slum versus non-slum gap has declined only marginally. A recent study demonstrated that multi-dimensional factors contributed to nutritional status of children under-five in urban Bangladesh—it found that a range of individual-, household-, and community-level factors affected stunting levels among under-five children living in the urban slums, whereas only individual level characteristics (e.g., mother’s age and education, child’s age, exposure to media) and the household’s socioeconomic status significantly affected stunting levels among children living in non-slums [31]. A review of ongoing programs also indicates a gap in child nutrition services in urban areas [16, 32]. Particularly, the PHC package implemented by the NGOs does not contain crucial elements of child nutrition services, like community-based nutrition services [33]. Evidence from the literature indicates that nutrition interventions have been marginalized relative to basic maternal health and family planning objectives, and nutrition services were found lacking in all private and NGO facilities who provide services to urban poor [17, 34, 35]. For example, BRAC’s Manoshi program only focuses on maternal nutrition counseling and health education on exclusive breastfeeding as a part of the essential newborn care [36]. Though USAID/DFID supported NHSDP’s nutrition interventions include nutrition counseling for mothers and promoting infant and young child feeding practices, USAID’s 2-year audit of NHSDP found that the program lacked comprehensive plan to improve technical capacity of NGOs and clinic staff, which include nutrition interventions [37].

A major limitation of the studies on intra-urban inequity is identification of slum settlements. In the UHS rounds, we followed the UN-Habitat definition of a slum: settlements with a minimum of 10 households or a mess unit with a minimum of 25 members, and predominantly very poor housing, very high population density, very poor water and sanitation, very low socioeconomic status, and lack of secure tenure to identify slum settlements [38]. The 2006 UHS identified slum settlements through a complete mapping and census of slums in the city corporations [7] and the 2013 UHS only mapped slums for a random sample of areas (specifically Mohallas, defined as urban communities) within the city corporations rather than doing a full census of slums [18]. Neither of the UHS rounds identified slums settlements outside the city corporations. Another limitation of this analysis is using the number of household assets as a proxy for household’s socioeconomic status—though household assets provide a good proxy for a household’s long-run wealth compared to relative indices or information on income/expenditure in low- and middle-income settings [39, 40], price of all the assets considered in the analysis were not similar and hence different combination of assets may indicate different socioeconomic status. Lastly, we chose the DID method for this analysis as it relies on a less strict exchangeability assumption and therefore is used in observational settings. DID requires data from pre-/post-intervention or repeated cross-sectional data to remove biases between the groups that could be the result from permanent differences between those groups. In the absence of pre-trend data, the findings from the DID model must be interpreted with caution [20].

## Conclusions and Way Forward

Given the existing trends in urbanization, both the slum and non-slum population are expected to grow rapidly in the coming decades. In order to cater needs for health, family planning and nutrition services of urban population, particularly of the urban poor living in slums, specially designed and well-targeted interventions are required to improve health equity. The rural-urban difference in family planning, maternal and child health, and nutrition in Bangladesh has been comprehensively explored and well documented [29, 41–44]. However, nationally representative figures to portray intra-urban inequity in Bangladesh, and potential drivers to affect the intra-urban gap, were not available and this paper fills an important gap in the literature on equity in urban settings.

The Government of Bangladesh’s 7th Five Year Plan for 2016–2020 and Urban Health Strategy 2014 emphasized improving the conditions of urban populations and reduce urban inequity in the coming years [14, 24]. On this backdrop, we propose the following policy and programmatic actions to be adopted to ensure equitable coverage of basic health services in the urban areas:

1. Capitalize on the progress made in reproductive health among the urban poor: Building on the progress made in contraceptive uptake and utilization of basic reproductive health services in urban slums [15, 45–47], establishing mechanisms to improve the contraceptive method mix towards more effective methods (i.e., long-acting reversible contraceptives and permanent methods), strengthening capacity of NGOs to expand SBA coverage in urban areas, and introducing innovative financing mechanisms like demand-side financing schemes will result in further narrowing of intra-urban differentials in the coming years [48–50].
2. Continue and expand community-based health services in urban areas: Continue the existing community-based basic health and FP services in the urban areas by the NGOs and expand its focus on delivering nutrition as well as behavior change communication services with a focus on slum populations. In order to achieve this, capacity development and appropriate incentives (both financial and non-financial) for CHWs [51–55] and efficient supply side management are crucial [45, 48].
3. Establish effective coordination mechanism between the MOHFW and the MOLGRDC: To deliver a comprehensive PHC package (that includes delivery care, family planning, and nutrition services) for the urban population, the government needs to take additional steps to forge a strong partnership between MOHFW and MOLGRDC to ensure adequate human resources and supplies for urban areas. The draft guideline and protocols for the referral system between PHC providers in urban areas and secondary-/tertiary-level health facilities also need to be endorsed and implemented [14].
4. Measure intra-urban differences in regular intervals: Given the scope of the existing routine health information system and household surveys in the health sector, and their limitation to measure health outcomes and service utilization status by the urban domains (viz. slums and non-slums), specially designed Urban Health Surveys should be taking place periodically to monitor health equity and differentials among urban groups. This would be crucial to monitor the coverage of outreach health and nutrition services in urban areas and to identify vulnerable pockets where services are not reaching.
5. Strengthen the government’s stewardship role in urban health: In order to ensure availability of resources, quality of care and affordability in urban health services, the government needs to carry out its stewardship functions effectively. These include, among others, efficient management of NGO contracting to deliver urban health services [15, 56]; increase resources from domestic as well as international sources for delivering a comprehensive urban health package; and strengthen regulatory capacity to ensure quality of services particularly in the private sector [34, 57].
